# Kitamura Electrophilic Fluorination Using HF as a Source of Fluorine

**DOI:** 10.3390/molecules25092116

**Published:** 2020-04-30

**Authors:** Jianlin Han, Greg Butler, Hiroki Moriwaki, Hiroyuki Konno, Vadim A. Soloshonok, Tsugio Kitamura

**Affiliations:** 1 Jiangsu Co-Innovation Center of Efficient Processing and Utilization of Forest Resources, College of Chemical Engineering, Nanjing Forestry University, Nanjing 210037, Jiangsu, China; 2 Oakwood Chemical, Inc. 730 Columbia Hwy. N, Estill, SC 29918, USA; gbutler@oakwoodchemical.com; 3 Hamari Chemical Ltd., 1-4-29 Kunijima, Higashi-Yodogawa-ku, Osaka 533-0024, Japan; hiroki-moriwaki@hamari.co.jp; 4 Department of Biological Engineering, Graduate School of Science and Engineering, Yamagata University, Yonezawa, Yamagata 992-8510, Japan; konno@yz.yamagata-u.ac.jp; 5 Department of Organic Chemistry I, Faculty of Chemistry, University of the Basque Country UPV/EHU, Paseo Manuel Lardizábal 3, 20018 San Sebastián, Spain; 6 IKERBASQUE, Basque Foundation for Science, Plaza Bizkaia, 48011 Bilbao, Spain; 7 Department of Chemistry and Applied Chemistry, Saga University, 1 Honjo-machi, Saga 840-8502, Japan; kitamura@cc.saga-u.ac.jp

**Keywords:** electrophilic fluorination, hydrogen fluoride, hypervalent iodine, fluorination reagents, bioactive compounds

## Abstract

This review article focused on the innovative procedure for electrophilic fluorination using HF and in situ generation of the required electrophilic species derived from hypervalent iodine compounds. The areas of synthetic application of this approach include fluorination of 1,3-dicarbonyl compounds, aryl-alkyl ketones, styrene derivatives, α,β-unsaturated ketones and alcohols, homoallyl amine and homoallyl alcohol derivatives, 3-butenoic acids and alkynes.

## 1. Introduction

**Figure 1:**
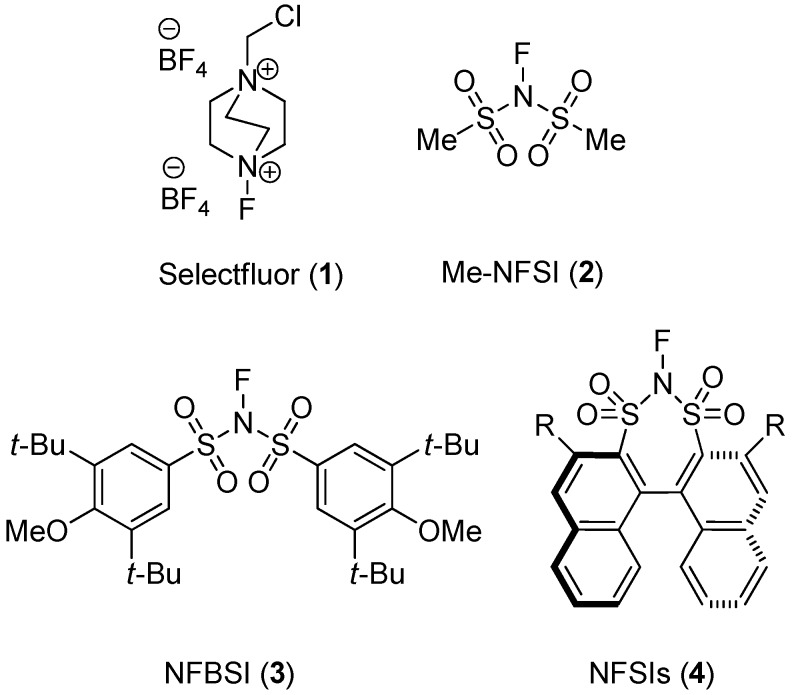
Some commercially available N–F electrophilic fluorination reagents.

Over the last two decades, the chemistry of fluorine-containing compounds has emerged as one of the exciting areas of multidisciplinary research. The most notable impact of fluorine can be seen in materials [1,2,3,4,5,6,7,8,9,10], agriculture [11,12,13] and health-related industries [14,15,16,17,18,19,20,21,22]. To sustain the continuous advancement and pace of the innovations enabled by fluorine, many research groups are focusing on new methodological inventions allowing for more selective and economical syntheses of structurally diverse fluoro-organic compounds [23,24,25,26,27,28]. For instance, the recent progress in asymmetric synthesis of fluorine-containing tailor-made amino acids [29,30,31,32,33,34,35,36,37,38,39,40,41,42,43,44,45,46,47,48] was stimulated by growth in their applications to drug design [49] magnetic resonance imaging [50,51], positron emission tomography [52,53], and peptide/protein engineering [54,55,56,57,58,59,60]. Nevertheless, while some complex polyfunctional fluorine-containing molecules possessing useful properties represent the ultimate target of synthetic chemistry, more fundamental research still focuses on the formation of the C–F bond. In this regard, electrophilic fluorination is one of the most pioneering and rapidly developing areas of study [61,62,63,64,65,66,67]. Conceptually, this approach requires a carbon-centered nucleophile and an electrophilic source of fluorine. While the former is a well-established chemical unit, the “electrophilic fluorine” is still a rather exotic and mechanistically controversial entity [68,69,70,71]. The major thrust of research activity in this field was centered on the development of the corresponding reagents capable of releasing the required “electrophilic fluorine”. The most successful results have been achieved utilizing compounds with N–F bonds. For example, as presented in Figure 1, 1-chloromethyl-4-fluoro-1,4-diazoniabicyclo[2.2.2]octane bis(tetrafluoroborate) (**1**), introduced by Professor E. Banks [72], also known as Selectfluor, is one of the best reagents used as a source of “electrophilic fluorine”. Other reagents, developed by Professor N. Shibata [73,74,75,76], are *N*-fluoro-*N*-(methylsulfonyl)methanesulfonamide (Me-NFSI) (**2**), 3,5-di-*tert*-butyl-*N*-((3,5-di-*tert*-butyl-4-methoxyphenyl)sulfonyl)-*N*-fluoro-4-methoxybenzenesulfonamide (NFBSI) (**3**) and axially chiral NFSIs (**4**), which can be used for enantioselective fluorination [77]. All these reagents are shelf-stable, easy to handle and operationally convenient to use for various synthetic applications [78,79,80,81]. However, the preparation of N-F reagents usually requires molecular fluorine [72,82], rendering them rather expensive and not practical for large-scale syntheses.

From the standpoint of practicality, the application of HF, as a source of fluorine, with the in situ formation of the electrophilic species, would offer an attractive alternative as a general methodology for “electrophilic” formation of a C–F bond. In this review article, we discuss the use of hypervalent iodine compounds, as electrophilic centers, and HF as a source of fluorine, creatively assembled in a one-pot sequence, allowing one to perform “electrophilic fluorination” of various types of organic compounds. The synthetic generality and practicality of these methods is critically discussed.

## 2. Fluorination of 1,3-Dicarbonyl Compounds

### 2.1. Iodosylbenzene-Mediated Fluorination

**Scheme 1:**
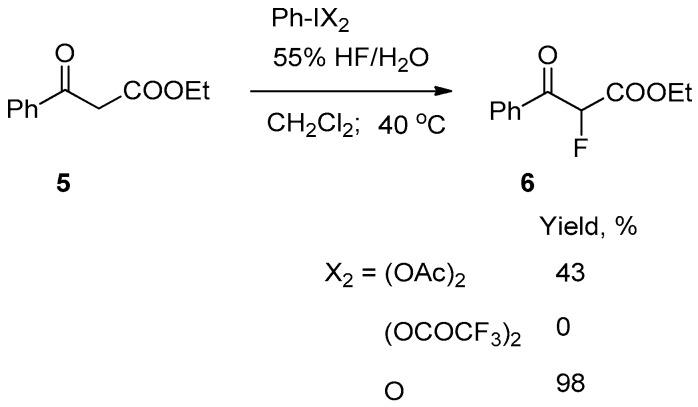
Fluorination of 3-oxo-3-phenylpropionate **5** using HF as a source of fluorine.

In 2011, Professor T. Kitamura’s group has reported that mixing ethyl 3-oxo-3-phenylpropionate (**5**) with 1.2 equivalents of a hypervalent iodine compound and excess (10-fold) of 55% aqueous HF resulted in formation of the corresponding ethyl 2-fluoro-3-oxo-3-phenylpropionate (**6**) in up to 98% chemical yield (Scheme 1) [83].

It was found that the nature of a hypervalent iodine compound played a key role in the reaction, affording an excellent yield of 98% of product **6** with the application of iodosylbenzene (PhIO). The outcome of this reaction, the substitution of acidic hydrogen in **5** by fluorine, is classified as “electrophilic” fluorination and was previously reported using F_2_ [84], XeF_2_ [85,86,87] fluoroxy compounds [88,89,90,91,92,93,94], N–F compounds [95,96,97,98,99,100,101,102] of type 1–4 (Figure 1) and (difluoroiodo)toluene [103,104]; all of them are typical electrophilic fluorination reagents.

**Scheme 2:**
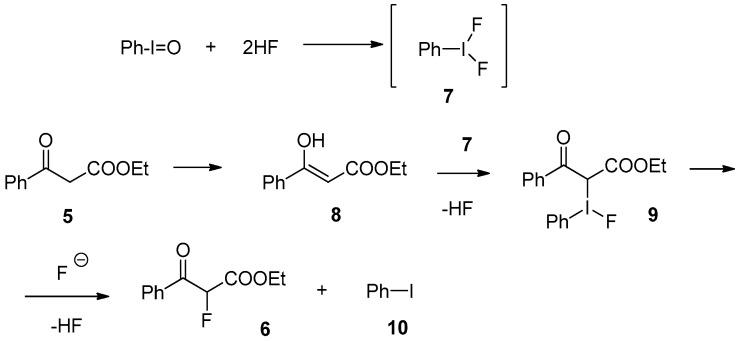
Mechanistic rationale of the “electrophilic” fluorination of 3-oxo-3-phenylpropionate **5** to 2-fluoro-3-oxo-3-phenylpropionate **6**.

The mechanistic rationale for the reaction is presented in Scheme 2. It postulates the in situ formation of (difluoroiodo)benzene **7** from iodosylbenzene and two equivalents of hydrogen fluoride. The reaction of **7** with the enol **8** gives rise to intermediate **9**, followed by the nucleophilic substitution of the iodine species to afford fluorinated compound **6** along with iodobenzene **10** as the final reaction products.

**Scheme 3:**
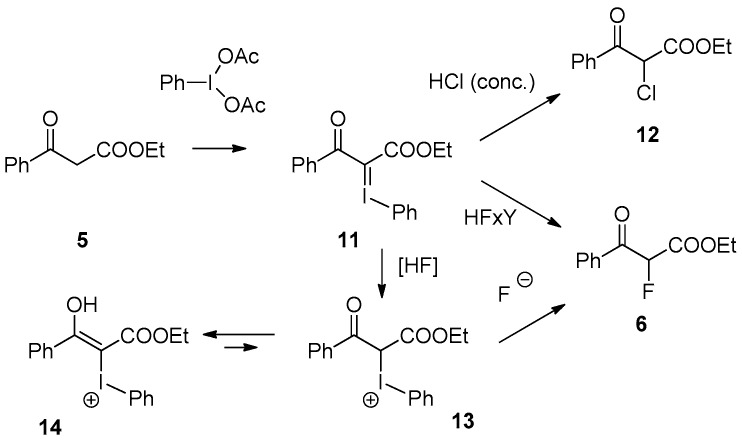
Synthesis of iodonium ylide **11** and its reactions with HF and HCl.

Some support for the proposed mechanistic pathway can be derived from the reactions of iodonium ylides with HF and HCl [105]. As shown in Scheme 3, iodonium ylide **11** can be prepared in quantitative yield by the reaction of keto ester **5** with (diacetoxyiodo)benzene in the presence of KOH in MeCN [106].

The reaction of iodonium ylide **11** with concentrated aqueous HCl, conducted in dichloromethane at ambient temperature, gave chlorinated product **12** in 55% yield [105]. Similar yields of fluorination product **6** were also obtained under the same conditions in the reactions of **11** with various HF reagents, such as 55% aqueous HF and TEA·3HF [105]. The noticeably lower chemical yields of **6** obtained in the reactions of iodonium ylide **11** with HF reagents (vs 98%), as compared with the reactions of **5** with HF in the presence of PhIO (Scheme 1 and Scheme 2) can be explained by enolization of intermediate **13** to vinyliodonium salt **14**, further reactions of which give complex mixtures of products.

**Scheme 4:**
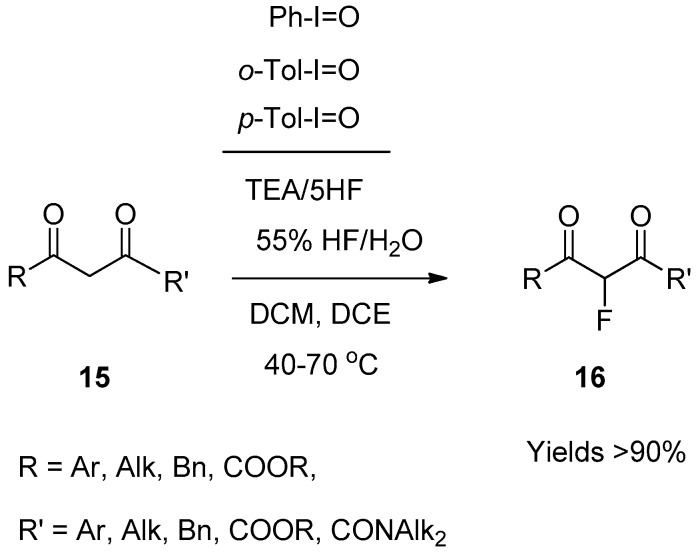
General synthetic applications of HF/PhIO for “electrophilic” fluorination of 1,3-dicarbonyl compounds.

Meticulous investigations of HF/PhIO reactivity revealed that this new approach has a general synthetic application for fluorination of various compounds bearing the acidic CH_2_ moiety, such as 3-keto-esters, 1,3-diketones and malonic acid derivatives, summarized in Scheme 4.

Optimization of the reaction conditions and reagents revealed that besides the original iodosylbenzene, its derivatives, such as *p*- and *o*-iodosyltoluene, can also be used successfully, and in some cases, even give better chemical yields as compared with unsubstituted iodosylbenzene [107,108]. For example, fluorination of amide derivatives of 3-keto-esters **15** (R’ = CONAlk_2_) with HF/PhIO affords the corresponding products **16** with relatively low (<50%) yield, while the application of *o*-iodosyltoluene allows for improved yields of the target products **16** (up to 93%). The source of the HF has also been examined in detail showing that other HF reagents such as complexes with triethylamine and pyridine can be successfully used in place of aqueous HF. Of particular importance is the wide synthetic generality of this approach. Thus, 3-keto esters **15** bearing an aromatic ring with electron-withdrawing or donating substituents can be successfully used as substrates. In the case of derivatives **15** featuring alkyl groups (R and/or R’ = Alk) the chemical yields of the corresponding fluorinated products **16** are a bit lower (~50–70%), likely due to possible enolization.

### 2.2. Catalytic Iodoarene-Mediated Fluorination

**Scheme 5:**
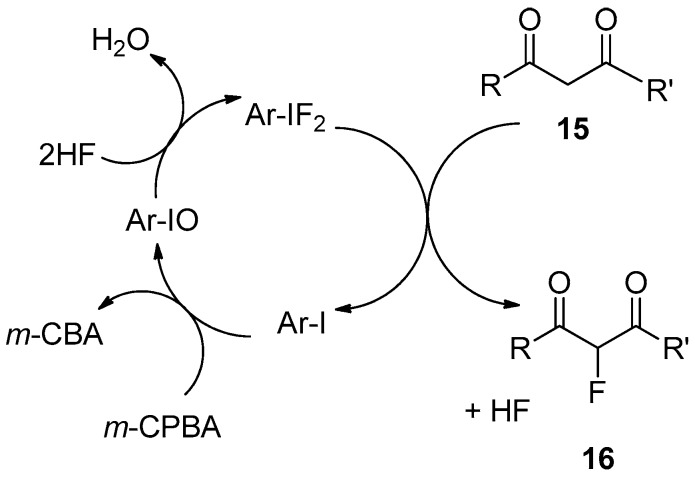
Proposed catalytic cycle of the iodoarene-mediated “electrophilic” fluorination.

Considering the postulated reaction mechanism (Scheme 2) involving eventual transformation (reduction) of iodosylbenzene to iodobenzene **10**, the final reaction product, the authors posited that providing in situ efficient oxidation of the latter to iodosylbenzene, would allow for a catalytic version of this process. Indeed, a significant breakthrough was made with the application of *m*-CPBA as a terminal oxidant [109]. As presented in Scheme 5, the Ar-I, used in catalytic amounts, undergoes a three-step transformation: oxidation to Ar-IO, reaction with HF to produce Ar-IF_2_ and reaction with the enolate form of **15** giving rise to the target product **16**.

**Scheme 6:**
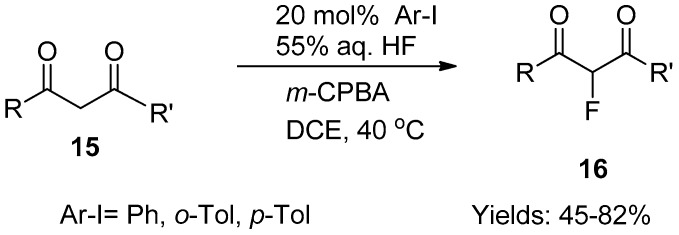
“Electrophilic” fluorination using a catalytic amount of Ar-I.

The optimized conditions for this catalytic “electrophilic” fluorination method are presented in Scheme 6 and included the following: as low as 20 mol% ArI, 55% aq. HF as a source of fluorine, *m*-CPBA as the oxidizing reagent and 1,2-dichloroethane as a solvent. The reactions are conducted at 49 °C and can be easily scaled up. The overall synthetic generality of this catalytic process is rather similar to the stoichiometric version.

The catalytic version can be used for fluorination of 3-keto-esters and 1,3-diketones bearing substituted aromatic, aliphatic, ester as well as amide groups. It should be notated, however, that the yields of target products **16** are a bit lower as compared with the results obtained in the reactions using a stoichiometric amount of the corresponding iodoarene. Furthermore, in most of the cases, application of *o*-Tol-I gave the best conversion of the starting 1,3-dicarbonyl compounds **15** and highest yields of fluorinated products **16**.

## 3. Fluorination of Aryl-Alkyl Ketones

Synthesis of fluorinated derivatives of monocarbonyl compounds, such as ketones and aldehydes, are of high importance in fluoro-organic chemistry [110,111,112,113,114,115,116,117,118,119,120,121]. These types of fluorinated derivatives are of proven synthetic value as building blocks for the preparation of a variety of polyfunctional fluorine-containing compounds of biological interest [30,122,123,124,125,126,127,128]. In particular, these derivatives can be easily transformed to the corresponding fluorinated amines and amino acids via biomimetic transamination [129,130,131]. General approaches for the preparation of α-fluoro-ketones include the following: two-step sequence of halogenation followed by nucleophilic substitution using fluoride [132,133,134,135,136] or a two-step process, involving the generation of the corresponding enolates, followed by fluorination using typical electrophilic fluorination reagents [137,138,139].

**Scheme 7:**
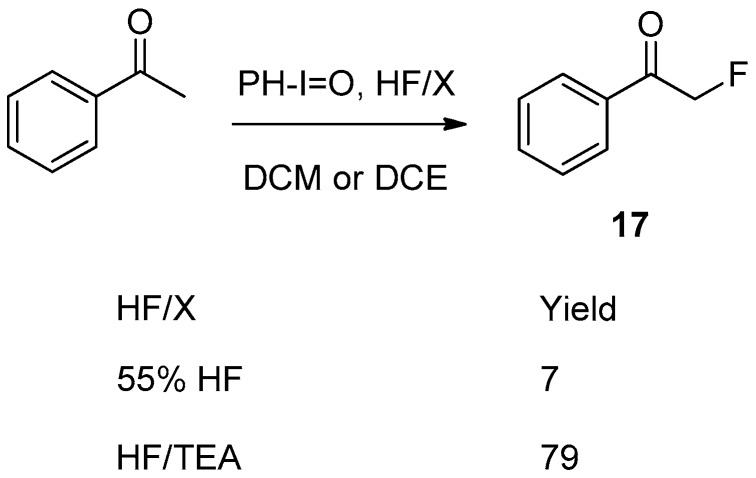
Fluorination of acetophenone.

It was found that the direct application of Kitamura’s approach, using PhIO/hydrofluoric acid, for fluorination of acetophenone (Scheme 7) gives very low yields of the target product **17**.

**Scheme 8:**
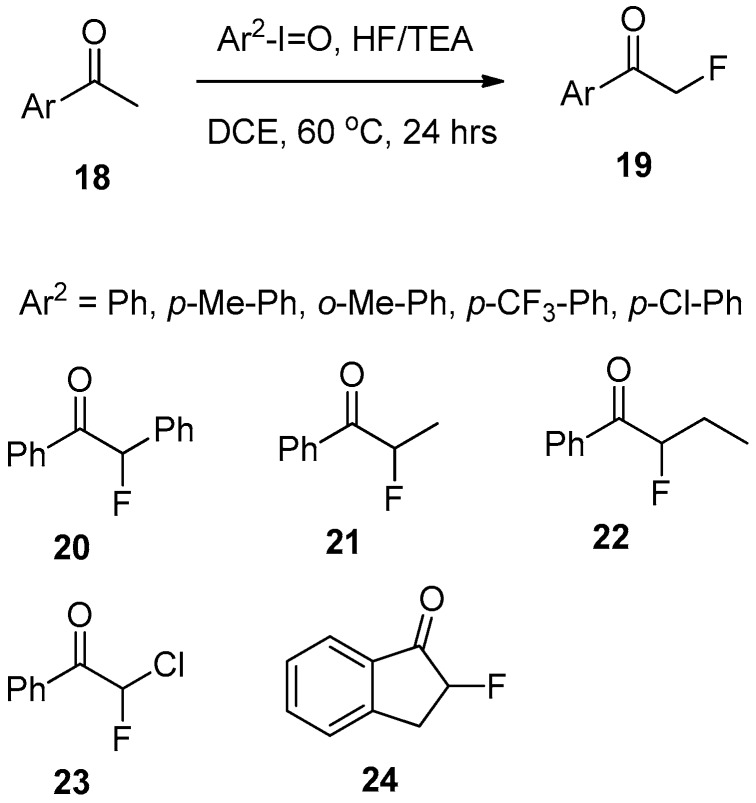
Substrate generality of the fluorination of ketones.

However, careful examination of various reaction conditions and reagents allowed for the discovery that the presence of water is detrimental for the fluorination process. The most likely reason is a reduction of fluoride’s nucleophilicity by coordination with water molecules. Consequently, it was found that the application of a triethylamine/5HF complex as a fluorine source provides for a smooth fluorination process affording α-fluoro-acetophenone **17** in a good yield [140]. Further research revealed that this reaction can be generally applied for various aryl-alkyl ketones (Scheme 8). Among possible sources of hypervalent iodine, *p*-iodosyltoluene (4-MeC_6_H_4_IO), *p*-chloloiodosylbenzene (4-ClC_6_H_4_IO), and *p*-iodosyl(trifluoromethyl)benzene (4-CF_3_C_6_H_4_IO) gave generally good results. Optimized reaction conditions included TEA/5HF in DCE (1,2-dichloroethane) at 60 °C for 24 h. Under these conditions, chemical yields of aryl-fluoromethyl ketones **19** varied between 70–85%. Of particular interest is the application of this reaction for α-fluorination of substrates derived from aryl-benzyl ketone **20**, higher alkyl derivatives such as **21** and **22**, haloalkyl **23** and cyclic compounds **24**.

**Scheme 9:**
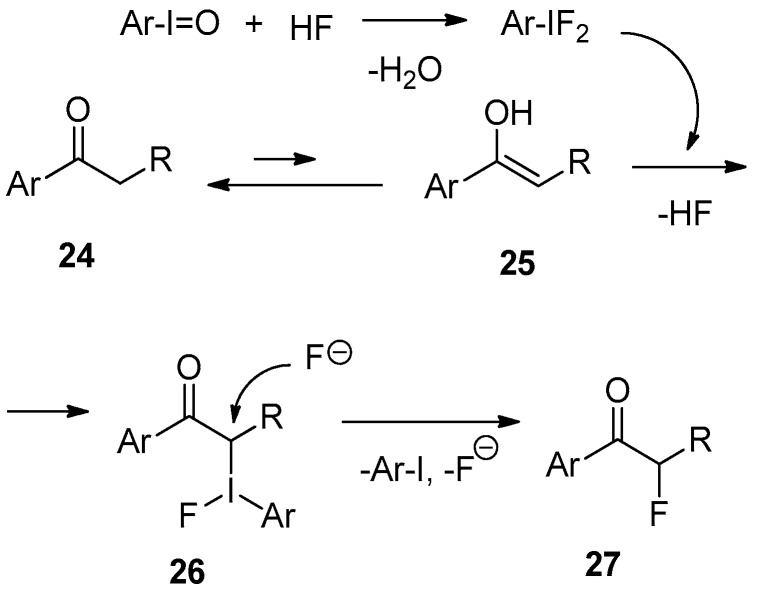
Proposed mechanism for electrophilic fluorination.

Mechanistically, the process can be envisioned as presented in Scheme 9. There are two first independent steps including in situ formation of Ar-IF_2_ from HF and Ar-I=O, and enolization of the starting ketone **24**. Electrophilic attach of Ar-IF_2_ on enolate **25** results in intermediate **26**, which is subjected to nucleophilic substitution with a fluoride ion giving rise to the final product **27**. The net result of this reaction sequence is the “electrophilic fluorination” of **24** to **27**.

## 4. Fluorination of Styrene Derivatives

In sharp contrast to a trifluoromethyl group, the CHF_2_ group is still rather scarcely represented among marketed pharmaceuticals [14,15,16,17,18,19,20,21,22]. Known biological properties of difluoromethyl-containing compounds [141], clearly suggest that the application of this fluorinated motif might be as successful as that already established for the trifluoromethylated compounds. The major reason for the current significantly lesser application of a CHF_2_ group in drug design is, most definitely, a lack of synthetic methods allowing for convenient installation of this functionality. Thus, most generally used methods include reaction of organozinc reagents with potassium bromodifluoroacetate [142], fluorination of gem-bistriflates and gem-dihalides [143,144,145,146], fluorodecarboxylation of dicarboxylic acids [147,148] and chlorodifluoromethylation followed by the elimination of HCl and migration of the double bond [149].

### 4.1. Hypervalent Iodine-Mediated Fluorination

#### 4.1.1. Stoichiometric Hypervalent Iodine Reagents

**Scheme 10:**
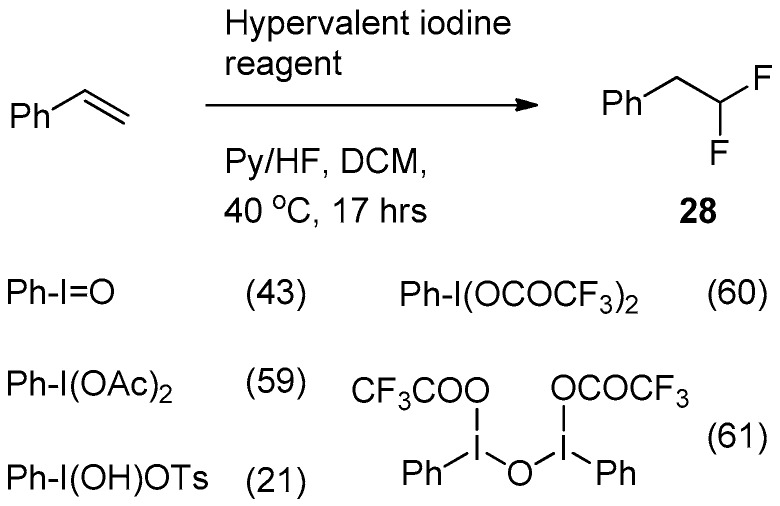
Fluorination of styrene.

The synthesis of difluoromethyl-containing compounds via reaction of styrene derivatives with fluorinated hypervalent iodine reagents or iodine in the presence XeF_2_ [150,151,152,153,154,155,156] is also a known method. However, the necessity of preparing hypervalent iodine compounds or the use of XeF_2_ limits its synthetic applications. With this in mind, Kitamura’s approach was examined for the fluorination of styrene substrates [157]. As presented in Scheme 10, a series of hypervalent reagents were screened and the trifluoroacetoxy derivatives were identified as the best.

It should also be noted that a separate optimization study showed that a complex of HF with pyridine was found to serve as a superior source of nucleophilic fluoride. Thus, under these optimized conditions, the target fluorinated product **28** was prepared in greater than 60% yield [157].

**Scheme 11:**
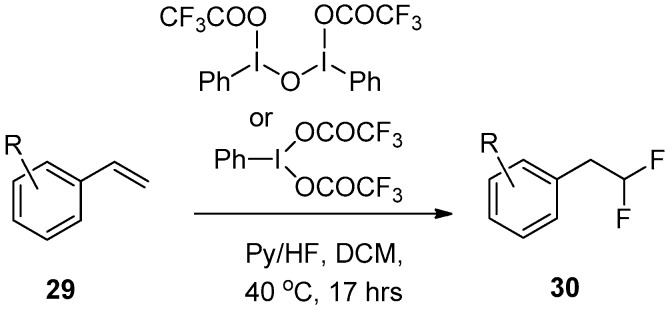
Generality of styrene fluorination using hypervalent iodine and HF complex with pyridine.

Application of these reaction conditions for fluorination of various substituted styrenes gave rather good results. This approach was applied to substrates **29** (Scheme 11) bearing alkyl, halo and OAc-type substituents in the *o*-, *m*- or *p*-position on the phenyl ring. The chemical yields of difluorinated products **30** ranged from 50% to 93%.

**Scheme 12:**
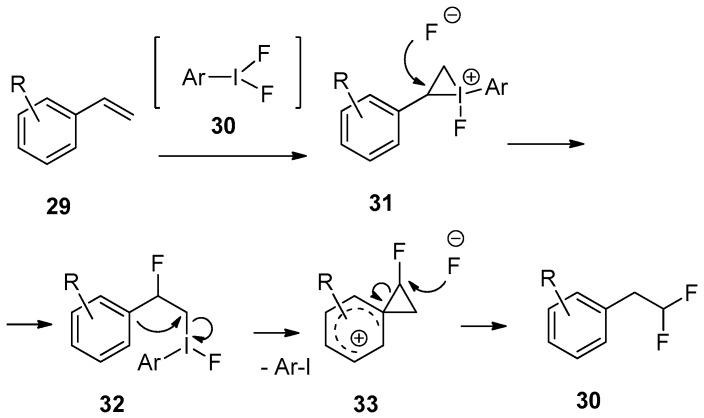
Mechanism of styrene fluorination.

Some structural limitations of this method may arise from a mechanism involving the migration of an aryl group. As presented in Scheme 12, in situ generated electrophilic hyper-iodide reagent **30**, reacts with starting styrene **29** to form three-ring intermediate **31** which is opened with fluoride to afford mono-fluorinated compound **32**. The latter proceeds to a second spirocyclic three-ring intermediate **33** with the elimination of Ar-I. The final step of the process is the nucleophilic opening of **33** with fluoride to give the difluorinated product **30**. One can assume that the stability of intermediate **33** will be strongly influenced by the electronic properties of the substituents on the aromatic ring. Nevertheless, as mentioned above, moderately electron-donating/withdrawing substituents, such as alkyl groups and halogens, can be tolerated giving product **30** with synthetically attractive chemical yields.

#### 4.1.2. Catalytic Hypervalent Iodine Reactions

**Scheme 13:**
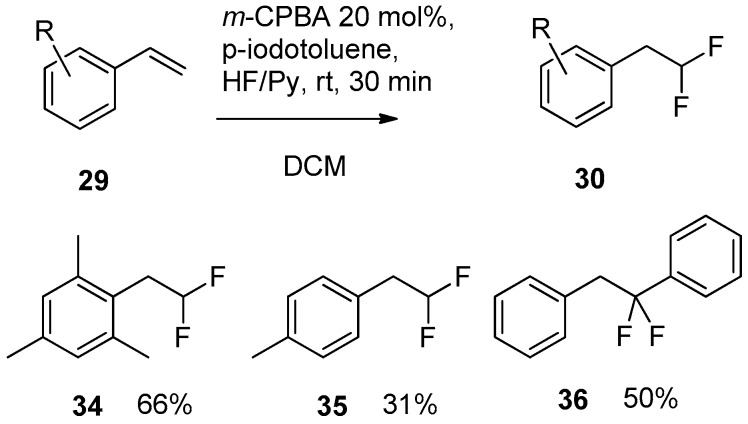
Catalytic version of styrene fluorination.

The catalytic version (Scheme 13) of this process was realized with application of 4-iodotoluene, HF/Py complex and *m*-CPBA, as the oxidizing reagent [157].

However, the outcome of the catalytic reactions was not entirely successful as difluoro products **30** were generally isolated in relatively low yields of about 50%. The highest yield (66%) in the series was obtained for the electron-rich trimethyl derivative **34**. In sharp contrast, mono-methyl substituted compound **35** was prepared in only 31% yield. It is interesting to note that fluorination of 1,1-diphenylethene resulted in rearranged difluoro compound **36** isolated in 50% yield.

## 5. Fluorination of α,β-Unsaturated Ketones and Alcohols

**Scheme 14:**
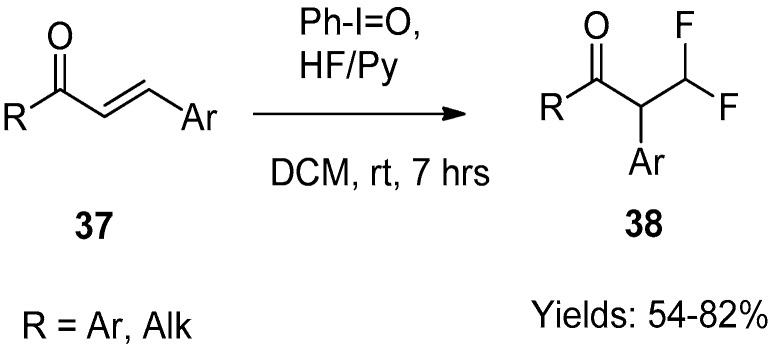
Fluorination of α,β-unsaturated ketones.

One may assume that a chemically similar process can also be realized for fluorination of other types of unsaturated compounds [158]. For example, as presented in Scheme 14, it was found that α,β-unsaturated ketones of general structure **37** can be converted to ketones **38** featuring α-aryl and difluoromethyl groups [159].

Substrate generality in this reaction is rather broad as the substituent R in starting **37** can be a methyl, long-chain alkyl, *tert*-butyl, aromatic or heteroaromatic group. Furthermore, the aromatic ring on the carbonyl carbon can bear electron-withdrawing or -donating groups, including NO_2_, halogens, Alk-O and Ac-NH. The aromatic group on the unsaturated C=C fragment is migrating during the reaction to the α-position, relatively to the carbonyl, and therefore is a bit more sensitive to the nature of substitution. Nevertheless, alkyl and halogen groups on the para position of the Ar moiety seem to be perfectly tolerated [159].

A catalytic version of this process was successfully realized using *m*-CPBA for the in situ oxidation of *p*-Tol-I to *p*-Tol-I=O. Optimized conditions included *p*-Tol-I (0.2 mol%), HF/Py (40 mol%) and (1.3 mol%). Substrate generality under the catalytic conditions was not compromised, however, the chemical yields were about 5–10% lower when compared with those obtained for the reactions conducted with stoichiometric amounts of the *p*-Tol-I=O [159].

**Scheme 15:**
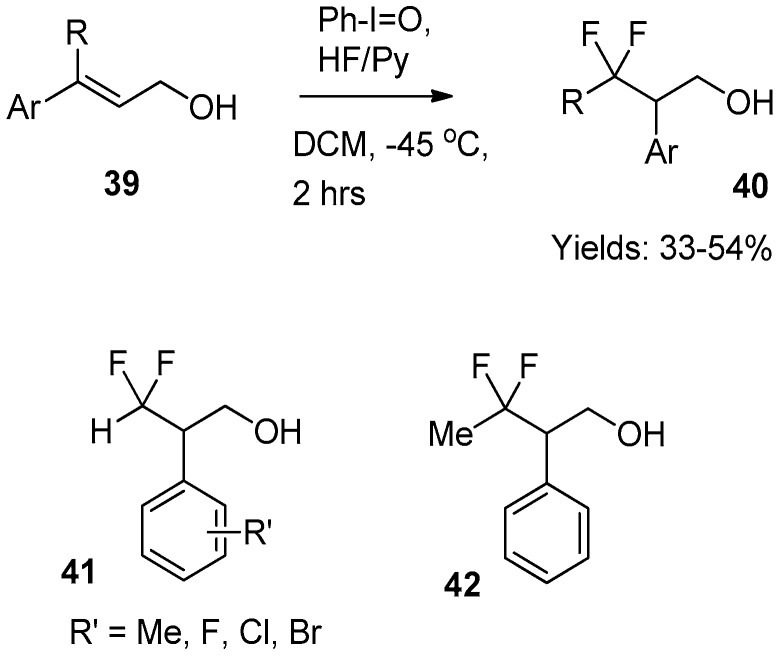
Fluorination of cinnamyl alcohol derivatives.

Of particular interest are the results reported for the fluorination of cinnamyl alcohol derivatives [160]. As presented in Scheme 15, starting compounds **39** were treated with Ph-I=O and HF/Py in dichloromethane to furnish fluorinated products **40**.

It should be noted that this type of fluorination required low reaction temperature due to the sensitive nature of the cinnamyl alcohol functionality. Similar to the previously discussed fluorination of compounds with conjugated C=C bonds, the reactions occurred with migration of the Ar group. Substrate generality study of these reactions was limited to two types of products, featuring difluoromethyl **41** and difluoroethyl groups **42**.

## 6. Cyclization–Fluorination Cascade

### 6.1. Homoallyl Amine Derivatives

**Scheme 16:**
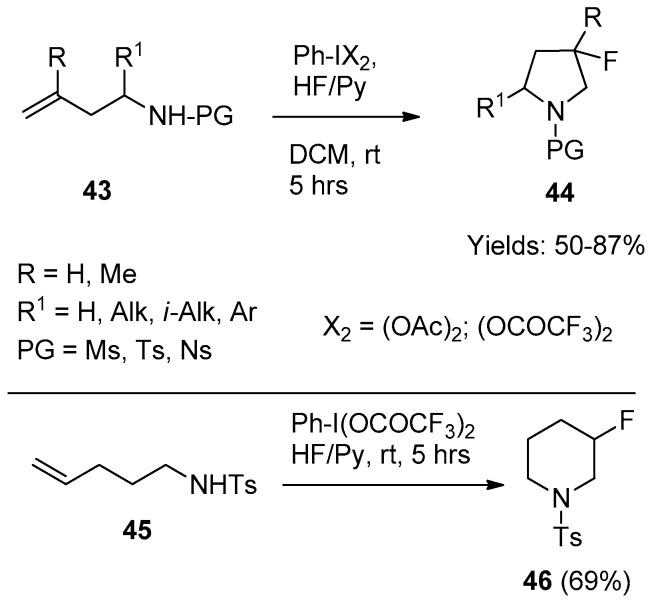
Intramolecular aminofluorination of homoallyl amine via cyclization-fluorination cascade.

Compounds containing the 3-fluoropyrrolidine moiety possessing a wide spectrum of biological properties. Some of them have been developed as dipeptidyl peptidase inhibitors [160,161,162,163], glucokinase activators [164] prolyl oligopeptidase inhibitors [165] and purine nucleoside phosphorylase inhibitors [166]. The most commonly used synthetic approach for preparation of 3-fluoropyrrolidines is based on fluorine substitution for hydroxy group in 3-hydroxylpyrrolidines [167,168,169,170,171]. Another approach, based on aminofluorination of alkenes [172,173,174,175,176], is more practical allowing both ring construction and introduction of a fluorine atom in one convenient synthetic sequence. It was found that the aminofluorination version of this approach can be successfully realized using the Kitamura fluorination protocol. As presented in Scheme 16, treatment of homoallyl amines **43** with a hypervalent iodine reagent and HF/Py afforded 3-fluoropyrrolidines **44** with respectable yields ranging from 50% to 87% [177].

Optimization of the reaction conditions in terms of hypervalent iodine reagent and source of HF found that a combination of HF/Py and PhI(OCOCF_3_)_2_ or PhI(OAc)_2_ was superior to other compounds such as PhI(OAc)_2_ and aqueous 55% HF and PhI(OH)OTs and HF/TEA complex [177]. As for the starting homoallyl amines, protection of the amino group with strong electron-withdrawing groups such as Ts, Ms or Ns, was found to be essential for the successful transformation. From the standpoint of generality, the process was shown to be applicable for a reasonably wide range of compounds with the substituent R bearing hydrogen or alkyl groups and with the R^1^ representing hydrogen, alkyl, bulky iso-alkyl and aryl groups. Quite remarkably, the reaction can be used for preparation of six-membered rings, as represented by the transformation of *N*-tosyl-4-pentenylamine **45** to *N*-tosyl-3-fluoropiperidine **46**, in 69% yield [177].

**Scheme 17:**
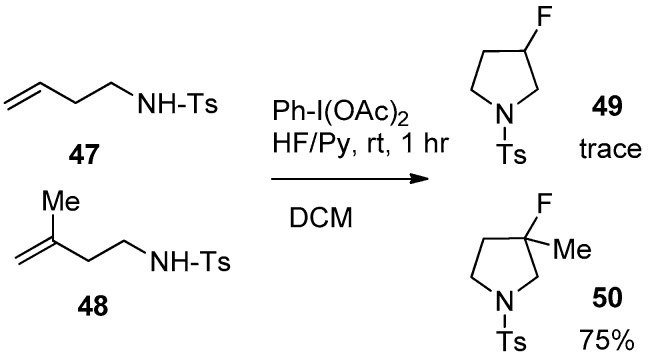
Competitive intramolecular aminofluorination.

To obtain information about the reaction mechanism for this intramolecular aminofluorination, the authors conducted a competitive reaction between homoallyl amines **47** and **48** (Scheme 17).

**Scheme 18:**
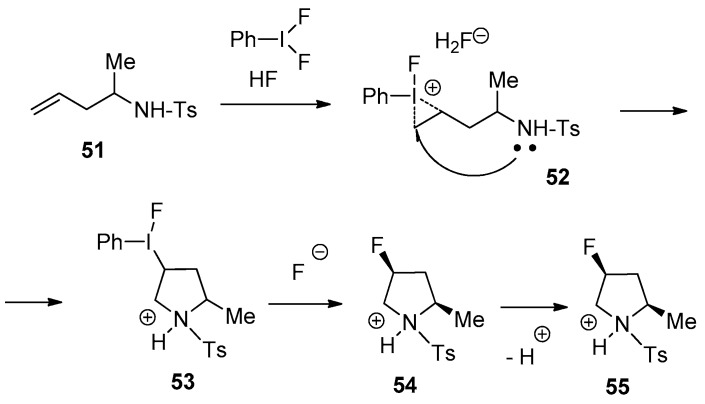
Mechanism of the intramolecular aminofluorination.

To this end, an equimolar mixture of **47** and **48** was subjected to the aminofluorination reaction using Ph-I(OAc)_2_ and HF/Py. It was found that, almost exclusively, homoallyl amine **48** was transformed to 3-fluoro-3-methylpyrrolidine **50**, while product **49**, derived from homoallyl amine **47**, was detected in the reaction mixture only in trace amounts. This result strongly suggested that the hypervalent iodine reagent preferentially reacts with the more electron-rich olefinic moiety. Based on the outcome of this competitive reaction, the authors proposed the following reaction mechanism, presented in Scheme 18.

According to the proposed mechanism, the formed in situ (difluoroiodo)benzene is activated by HF and interacts with the nucleophilic double bond of homoallyl amine **51** resulting in formation of the cyclic iodonium salt **52**. The three-membered ring is opened next by the nitrogen, attacking the terminal carbon by the bridged iodonium salts **52** to afford pyrrolidine **53**. The formation of intermediate **53** is followed by S_N_2 substitution by a fluoride ion. The final step is the deprotonation of **54** giving rise to the final 3-fluoropyrrolidine product **55**.

### 6.2. Homoallyl Alcohol and 3-butenoic Acid Derivatives

**Scheme 19:**
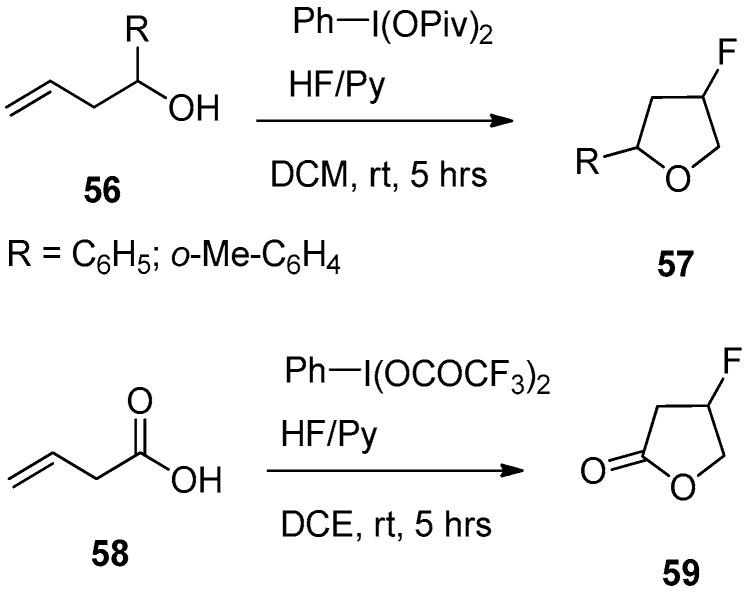
Fluorination of homoallyl alcohols and 3-butenoic acid derivatives.

An interesting extension of this reaction cascade was demonstrated for other types of polyfunctional olefins, such as homoallyl alcohols and 3-butenoic acid derivatives [178]. The fluorination-cyclization cascade of homoallyl alcohols **56** (Scheme 19) was performed using the reagent system of PhI(OPiv)_2_ and HF/Py in dichloromethane. The target fluorinated tetrahydrofuran derivatives **57** were isolated with reasonably good 54−65% yields as a mixture of cis- and trans-isomers.

Under similar conditions, except using Ph-I(OCOCF_3_)_2_ as the hypervalent iodine reagent and dichloroethane as a solvent, butenoic acid **58** was transformed to fluorinated butyrolactone **59** in 45% yield. The structure of products **57** and **59** is consistent with the above-discussed mechanism (Scheme 18) for the fluorination-cyclization cascade. Thus, in the cases of homoallyl alcohols **56** and 3-butenoic acid **58** the oxygen of the hydroxy group acts as the nucleophilic element attacking the terminal carbon of the corresponding intermediate bridged iodonium salts of type **52**, to complete the cyclization step.

## 7. Reactions with Alkynes

**Scheme 20:**
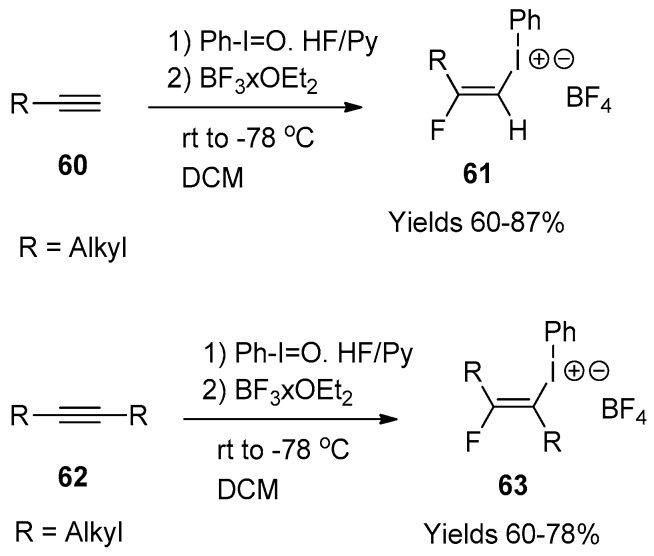
Synthesis of β-fluorovinyliodonium salts by the reaction of alkynes with hypervalent iodine/HF reagent.

One of the most recent developments in this chemistry is the synthesis of β-fluorovinyliodonium salts via the reaction of alkynes with hypervalent iodine reagents in the presence of HF (Scheme 20) [179].

**Scheme 21:**
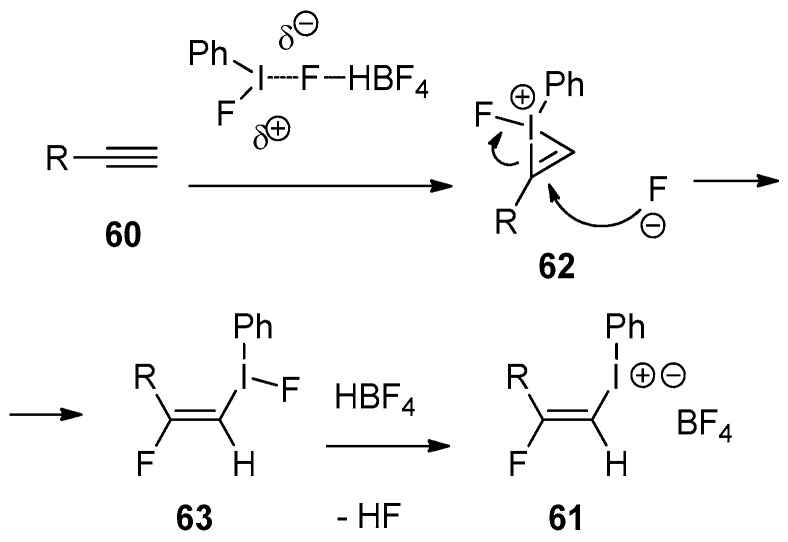
Proposed mechanism for the reactions of alkynes with Ph-I=O and HF/Py in the presence of BF_3_·OEt_2_.

It should be noted that direct application of the conditions developed for the reactions of various C=C unsaturated compounds was found to be ineffective for alkynes. In a series of preliminary experiments, it was determined that Ph-IF_2_ needed some additional activation by a Lewis acid, such as BF_3_·OEt_2_. Indeed, treatment of mono-alkyl substituted alkynes **60** with Ph-I=O and HF/Py, followed by the addition of BF_3_·OEt_2_, resulted in the formation of β-fluorovinyliodonium salts **61** with a respectable chemical yield. Compound **61** is quite stable to be isolated and fully characterized. In terms of generality one can mention that the alkyl group could bear in its terminal position some functionalities, such as aromatic or heterocyclic rings, a protected alcohol or ester group. The reactions are highly regio- and stereospecific as the fluorine being added to the most substituted carbon on **60** and products **61** are obtained as trans-isomers only. This set of conditions can also be successfully applied to symmetrically disubstitute alkynes **62**. The corresponding β-fluorovinyliodonium salts **63** were obtained exclusively as trans-isomers with 60–78% yields. The proposed mechanism for the reactions of Ph-I=O and HF/Py reagents with alkynes is presented in Scheme 21.

According to the proposed mechanism, the in situ generated PhIF_2_ is activated by HBF_4_ and undertakes the electrophilic addition to alkyne **60** affording bridged three-membered iodonium species **62**. Intermediate **62** is subjected to the nucleophilic attack of fluoride ions leading to the formation of (*E*)-β-vinyliodonium fluorides **63**. The final step in this sequence is the ligand exchange with HBF_4_ yielding final product **61**. It should be emphasized that the geometric configuration of **63** is controlled by the ring-opening of iodonium species **62** with fluoride ion.

## 8. Conclusions

The chemistry discussed in this review article is based on the original idea of the application of HF as a source of fluorine for subsequent “electrophilic” formation of a C–F bond. The target transformation is achieved via in situ generation of the proper electrophilic species derived from hypervalent iodine compounds. The data reported so far clearly show the great synthetic value of this approach for fluorination of various 1,3-dicarbonyl compounds, aryl-alkyl ketones, styrene derivatives, α,β-unsaturated ketones and alcohols, homoallyl amine and homoallyl alcohol derivatives, 3-butenoic acids and alkynes. The major advantage of this chemistry over alternative approaches is its practicality and very attractive cost-structure, boding well for its application in large-scale synthesis of important drug intermediates or other industrial fluoro-organics. However, there are still some issues that need to be solved, to further increase the synthetic value of this methodology. One critical area of improvement would be the application of more safe and still cheaper sources of HF. Thus, most of the research has been performed using aqueous HF, and its complexes with TEA and Py. One would also suggest HF in complex with THF as an alternative source, which was reported by Professor W. R. Dolbier [179,180]. Finally, further optimization of the reaction conditions, alongside with applications of new sources of HF, leading to increased chemical yield would clearly be in the focus for future research in this exciting area of fundamental fluoro-organic methodology. As a word of caution, we would like to remind the readers that hydrogen fluoride, aqueous or in complexes with pyridine, triethylamine or in any other form, is quite toxic, highly corrosive and can easily penetrate skin and muscles destroying cell membranes and nerves. The reactions should be conducted in a well-ventilated hood using Teflon-lined reactors of tubes.
